# Investigating the Molecular Mechanism of Aqueous Extract of *Cyclocarya paliurus* on Ameliorating Diabetes by Transcriptome Profiling

**DOI:** 10.3389/fphar.2018.00912

**Published:** 2018-08-09

**Authors:** Jing Li, Mei Luo, Minghua Hu, An-Yuan Guo, Xiangliang Yang, Qiong Zhang, Yanhong Zhu

**Affiliations:** ^1^National Engineering Research Center for Nano medicine, College of Life Science and Technology, Huazhong University of Science and Technology, Wuhan, China; ^2^Key Laboratory of Molecular Biophysics of the Ministry of Education, Department of Bioinformatics and Systems Biology, College of Life Science and Technology, Huazhong University of Science and Technology, Wuhan, China; ^3^Joint Laboratory for the Research of Pharmaceutics, Huazhong University of Science and Technology and Infinitus, Wuhan, China

**Keywords:** diabetes, pancreas islet, dyslipidemia, lipid metabolism disorders, transcriptome analysis

## Abstract

Diabetes is generally regarded as a metabolic disorder disease caused by various reasons, including pancreas islet injury and lipid metabolism disorders. The aqueous extract of *Cyclocarya paliurus* leaves (CPAE) was reported to be anti-diabetic. However, the possible molecular mechanisms have not been investigated. To elucidate the anti-diabetic effects of CPAE and the underlying potential mechanisms, we performed transcriptome profiling (RNA-Seq and miRNA-Seq) on the pancreas and liver from non-diabetic, diabetic and diabetic-CPAE rats. Our results demonstrated the CPAE could reduce excessive oxidative stress and inflammation in the pancreas, and maintain the balance of glucose and lipid metabolism in the liver. Transcriptome profiling and regulatory network analysis indicated that CPAE may ameliorate diabetes through improving β-cell survival and strengthening insulin secretion in the pancreas. Meanwhile, CPAE could improve impaired lipid metabolism and reduce excessive oxidative damage in the liver probably through miR-200/375-*Aldh1b1*/*Hps5*-*Hes1* co-regulatory network. Taken together, our biochemical experiments combined with transcriptome profiling showed that the effects of CPAE on anti-diabetes may work through protecting pancreatic β-cell, improving dyslipidaemia and lipid metabolism disorders.

## Introduction

Diabetes is a chronic metabolic disease mainly caused by insulin dysfunction and pancreatic β-cell failure (Buchanan et al., 2002). Most available anti-diabetic drugs have some limitations, such as severe side effects and high rates of secondary failure (Bodmer et al., 2008). Natural products from plant/herb have evolved over 1000s of years and been used as complementary approach for health (Dong, 2013), and considered as effective strategies and alternative medicine for diabetes management and chronic diseases (Lee et al., 2012; Yao et al., 2015). *Cyclocarya paliurus* (Batal.) as a traditional Chinese medicinal herb, has been widely used for the prevention and treatment of diabetes in China (Yao et al., 2015). *In vitro* and *in vivo* studies have demonstrated that leaves of *C. paliurus* have therapeutic effects on oxidation injury, diabetes and hyperlipidemia (Kurihara et al., 2003; Xie et al., 2015). *C. paliurus* extracts could decrease blood glucose and increase insulin levels in diabetic rats through suppressing β cell apoptosis by modulating MAPK and Akt pathways (Xiao et al., 2017). However, systemically investigating the molecular mechanisms how the aqueous extract of *C. paliurus* leaves (CPAE) prevents diabetes at the transcriptional and post-transcriptional levels are still lacking.

Next generation sequencing based transcriptome profiling (RNA-seq, small RNA-seq) analysis is a powerful approach for investigating potential molecular mechanisms underlying complex biological processes (Han et al., 2014; Zhang et al., 2016), and has been widely applied in diabetic researches (Baran-Gale et al., 2013). Transcription factors (TFs) and microRNAs (miRNAs), as 2 main transcriptional regulators, play crucial roles in multiple biological processes including development and disease (Xu et al., 2016). For example, the dysregulation of miR-29 influenced the glucose and lipid metabolism in skeletal muscle in diabetes (Massart et al., 2017), and the TF FoxO1 could regulate hepatic insulin sensitivity and lipid metabolism (Matsumoto et al., 2006). Significantly, miRNAs and TFs can mutually regulate and construct regulatory loops by co-regulating target genes, contributing to the progress of disease (Zhang et al., 2015a). However, the combination of transcriptome profiling and regulatory network analysis for exploring potential molecular mechanisms of *C. paliurus* on anti-diabetes was still lacking.

In this study, we proved that CPAE can ameliorate diabetes induced by high fat diet (HFD) and streptozotocin (STZ), and then performed transcriptome profiling (RNA-Seq and miRNA-Seq) on the pancreas and liver to investigate the underlying molecular mechanisms. Results showed that CPAE exerted potent anti-diabetic effects through protecting pancreatic β cell, improving dyslipidaemia and lipid metabolism disorders.

## Materials and Methods

### Preparation of the CPAE

The *C. paliurus* was obtained from Jiangxi Xiushui Miraculous Tea Industry Co. (Jiangxi, China). Leaves were air dried and ground into powder which was extracted with 10 volumes (v/w) of boiling distilled water for 2 h. After filtering, the residue was extracted with 10 volumes (v/w) of boiling distilled water for 1.5 h again. The above filtrates were collected, concentrated and dried for use. It was dissolved in physiological saline before use. The content of polysaccharides was 2.35 g/100g of CPAE, which was determined by phenol-sulfuric acid colorimetrictitration method. The content of total flavonoids was 3.34 g/100g of CPAE determined by aluminum chloride method.

### Animal Procedure and Tissue Preparation

Male Sprague-Dawley rats weighting about 200 g were provided by Hubei Province Center for Disease Control and Prevention. All animal studies were approved by the Animal Experimentation Ethics Committee of Huazhong University of Science and Technology, and carried out in compliance with guidelines approved by the Science and Technology Department of Hubei Province.

Rats were housed under temperature-controlled rooms (20–22°C) with a 12-h light-dark cycle. All animals were given ad libitum access to feed and water. After acclimation for 1 week, animals were randomly assigned into four groups: (1) Non-diabetic group (*n* = 6): considered as negative controls, rats received daily normal saline, (2) Diabetic group (*n* = 8): rats received daily normal saline, (3) Diabetic-CPAE (*n* = 8): rats received daily CPAE (200 mg/kg body weight) dissolved in saline solution, (4) Diabetic-MET (metformin) (*n* = 8): considered as positive controls, rats received daily MET (200 mg/kg body weight) dissolved in saline solution. Rats in the non-diabetic group were fed with a standard diet. The diabetic group, Diabetic-CPAE and Diabetic-MET groups were fed with a 60% HFD (Trophic Animal Feed High-tech Co., Ltd, China) and followed by a single intraperitoneal injection of STZ (Sigma, St. Louis, MO, United States) at 35 mg/kg body weight in 0.1 mol/L citrate buffer (pH 4.5) at the end of 7 weeks. An equal volume of vehicle was injected into the non-diabetic rats. It was preventive administration and all the groups were treated for 12 weeks at the start of the experiment. At the end of the experiment, all rats were anesthetized with pentobarbital sodium after a 12 h fast. Serum samples were collected and stored at −20°C. The liver and pancreas tissues were removed, rinsed with physiological saline, immediately frozen in liquid nitrogen, and then stored at −80°C.

### Measurement of Blood Glucose, Oral Glucose Tolerance and Insulin Levels

Fasting blood glucose (FBG) was measured in blood collected from the tail every 2 weeks. Levels of blood glucose were determined with a blood glucose meter (Accu-Check Active 1, Roche Pharmaceutical Ltd., Basel, Switzerland). An oral glucose tolerance test was performed before animals were sacrificed. Animals fasted overnight and then received oral administration of 2.5 g/kg glucose (Sigma, United States). Blood glucose level was determined at 0 (before glucose administration), 30, 60, and 120 min after glucose administration. Insulin levels both in the serum and pancreas were assayed using insulin ELISA Kit (Nanjing Jiancheng Bioengineering Institute, China).

### Biochemical Analysis

Plasma levels of free fatty acids (FFA), total cholesterols (TC), triglycerides (TG), high density lipoprotein cholesterols (HDL-c) and low density lipoprotein cholesterols (LDL-c) were measured with commercially available kits (Nanjing Jiancheng Bioengineering Institute, China). Levels of TNF-α and IL-6 were detected using commercial ELISA Kits (Cloud Clone Corp, China). Levels of malondialdehyde (MDA), activities of superoxide dismutase (SOD) and concentrations of glutathione were detected with corresponding Kits (Nanjing Jiancheng Bioengineering Institute, China). Activities of serum aspartate aminotransferase (AST) and alanine aminotransferase (ALT), levels of blood urea nitrogen (BUN) and creatinine (Cre) were measured using automatic biochemical analyzer in Wuhan general hospital of Guangzhou military (China).

### Histological Analysis and Immunofluorescence

Histological analysis was performed by Wuhan Google Biotechnology Co., Ltd. (China). We randomly selected liver, pancreas and kidney tissues of 3 rats to perform histological analysis in each group. For each sample, 5 islets were measured for calculating islet/pancreas area ratio, while 10 glomerular basement membranes were chosen to evaluate the thickness. Briefly, liver, pancreas and kidney tissues were removed and fixed in 4% paraformaldehyde. After that, tissues were dehydrated in graded series of alcohol (70, 80, 90, 95, and 100%) and made transparent with xylene, embedded in paraffin, sectioned in 5 μm thickness (RM2235 ccwUS, Leica microscopy system co., LTD. Germany). Next, tissue sections were dewaxed in xylene two times, and then in 100, 95, and 80% ethanol to elute xylene, rinsed with water. After that, the sections were stained with hematoxylin for 5 min and washed. Then sections were stained with eosin for 2 min and washed with water. Finally, all sections were sealed with neutral resins. All slides in the current study were examined under a light microscope (DM3000, Leica Microscopy System co., Ltd., Germany).

Cell proliferation for Ki67 staining was performed by Wuhan Google Biotechnology Co., Ltd (China). Briefly, paraffin-embedded tissues were sectioned into 5-μm-thick slices. The sections were deparaffinized by xylene and dehydrated by a graded series of ethanol (100, 85, 75%). After that, tissue sections were placed in the microwave oven for antigen retrieval with a repair box filled with EDTA antigen repair buffer (pH9.0). Primary antibodies were added and incubated overnight at 4°C. After washing the slices, they were incubated with corresponding secondary antibodies for 50 min at the room temperature with dark. Next, they were washed, incubated with DAPI for staining of the nucleus at room temperature for 10 min with dark. Finally, they were washed and sealed. The slides were examined under a fluorescence microscope.

### Library Preparation, mRNA and Small RNA Sequencing and Differential Expression Analysis

Total RNAs were prepared using TRIzol reagent (Invitrogen Life Technologies, Rochester, NY, United States). Libraries for RNA-Seq (Ribo-Zero) and miRNA-Seq were prepared according to the TruSeq protocol (Illuminia Truseq V3, United States). RNA-seq and small RNA-seq were performed by the Illumina Hiseq 2000 platform with read length 2 × 150 bp at BGI Bioinformatics Institute (Shenzhen, China). Low quality reads were removed by in-house scripts for small RNA-Seq and RNA-seq. For RNA-seq reads, discarding these reads: (1) reads less than 35 bp after adapter trimming; (2) reads with multiple N (>5 bases); (3) reads with low quality bases (quality value ≤ 5, ratio of low quality bases > 10%). For miRNA-Seq reads, trimming the adapters and removing these reads: (1) reads with N base; (2) reads with length (> 45 nt or <15 nt); (3) reads with low quality (mean quality < = 20). All raw sequencing data have been deposited in the genome sequence archive of Beijing Institute of Genomics, Chinese Academy of Sciences, gsa.big.ac.cn (accession no. CRA000791). The aligner for RNA-seq reads was hisat2 (version 2.05), and the downstream pipeline for expression estimation of genes was according to the protocol of hisat2-stringtie (v1.2.2)-ballgown (v1.99.3) workflow (Pertea et al., 2016). Bowtie was employed to align reads to genome/miRbase for miRNA analysis (version: 1.1.2). The known miRNAs were identified by miRBase V21 database (Griffiths-Jones et al., 2008). The unannotated reads in Rattus norvegicus reference genome (version 6.0) were used to predict novel miRNAs by miRDeep (An et al., 2013). The NOISeq package was used to conduct the differentially expression of miRNAs (DEMs) and genes (DEGs) (FDR < 0.05 and |fold change| > 1) (Tarazona et al., 2012). All subsequent analysis was based on the DEMs and DEGs.

### Real-Time qPCR

Primers for gene including DNA-damage-inducible transcript 4 (*Ddit4*), Fibroblast growth factor 21 (*Fgf21*), Insulin 1 (*Ins1*) and Insulin 2 (*Ins2*) were synthesized by TSINGKE Biological Technology (China), while primers for miRNAs were designed and synthesized by Sangon Biotech (China). The cDNAs were synthesized using PrimeScript^TM^ RT reagent Kit according to products manuals (Takara Biotechnology, Dalian). Then Real-time PCR experiments were performed using SYBR Premix Ex TaqTMII (TliRNaseH Plus) (Takara Biotechnology (Dalian) Co., Ltd. China) in a Bio-Rad C1000 detecting system (StepOnePlus_1). The expression levels of β-actin and U6 were regarded as reference for gene and miRNA, respectively. The fold changes were calculated by 2^ΔΔC_T_^ method.

### Visualization and Bioinformatics Analysis

The expression levels of DEMs and DEGs were visualized by gplots package in R language. The miRNA-TF-target regulatory networks were conducted based on DEGs and DEMs by our previous method (Lin et al., 2015). The gene list of TFs was downloaded from AnimalTFDB 2.0 (Zhang et al., 2015b). All networks were visualized by Cytoscape (version 3.4.0) (Kohl et al., 2011). KEGG pathways and GO terms were enriched by in-house script with hypergeometric test.

### Statistical Analysis

Significance level 0.05 and power 0.9 were employed to estimate sample number in each group. According to our experiment design and pre-experiments, difference between the means divided by the pooled standard deviation (Effect size) was calculated, which is greater than 2. The pwr.t.test function in R language was used to calculate sample number for each group and n = 6.386753 was obtained. The results were expressed as mean value ± standard error mean (SEM). Significant differences between mean values of different groups were determined by one-way analysis of variance (ANOVA) with SPSS. The least significance difference was used for *post hoc* statistical test. *P*-value < 0.05 was considered as significant. ^∗^*p* < 0.05, ^∗∗^*p* < 0.001, ^∗∗∗^*p* < 0.0001, ns represented no significance.

## Results

### CPAE Improves Insulin Levels and Glucose Homeostasis by Reducing Oxidative Stress and Inflammation

**FIGURE 1:**
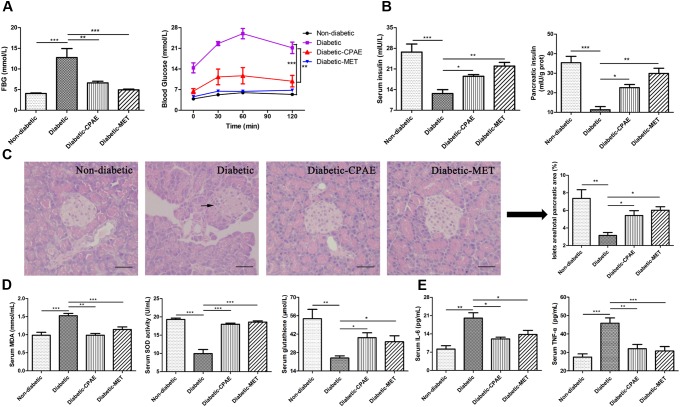
*Cyclocarya paliurus* leaves (CPAE) treatment alleviates diabetes in diabetic rats. **(A)** The level of FBG at end of experiment before sacrificed and levels of blood glucose before oral glucose and after glucose administration at 30, 60, and 120 min in glucose tolerance test. **(B)** Levels of insulin in the serum and pancreas tissue. **(C)** Histopathological examination of pancreas tissues (H&E staining) (magnification, 200×) and relative islets area in total pancreatic area. The scale bar is 40 μm. **(D)** Levels of MDA, SOD activities and glutathione concentrations in the serum. **(E)** Levels of TNF-α and IL-6 in the serum. All data are presented as means ± SEM (*n* = 6). ^∗^*p* < 0.05, ^∗∗^*p* < 0.001, ^∗∗∗^*p* < 0.0001.

To explore the protective and preventive roles of CPAE on diabetes, the CPAE was orally administrated for 12 weeks at the start of the experiment. HFD combined with low dose of STZ resulted in a drastic elevation of FBG level and oral glucose load (**Figure 1A**), this elevation was continually restricted by CPAE or MET treatment, and MET had a better hypoglycemic effect than CPAE (**Supplementary Figure S1A**). The body weight and food intake of diabetic rats were much lower than non-diabetic rats, and there is no significant difference between diabetic-CPAE and diabetic group (**Supplementary Figures S1B,C**). Compared with the diabetic rats, both CPAE and MET administration significantly increased insulin levels both in the serum and pancreas (**Figure 1B**). Meanwhile, histological examination revealed that islets were seriously damaged with irregular shape and reduced islet/pancreas area ratio in the diabetic group, while these characteristics were improved under CPAE administration (**Figure 1C**).

In addition, compared with the diabetic group, MDA levels were significantly decreased with CPAE or MET administration, while the SOD activities and glutathione concentrations were markedly increased, which may imply CPAE could relieve excessive oxidative stress (**Figure 1D**). Meanwhile, the levels of TNF-α and IL-6 were decreased in the diabetic-CPAE or diabetic-MET group (**Figure 1E**). These results suggested that CPAE could lower the blood glucose and increase the insulin levels probably through protecting pancreas islet from oxidative stress and inflammation damage, thus alleviating diabetes.

### Transcriptome Analysis Reveals That CPAE Protects Pancreatic β-cell to Increase the Insulin Levels

**FIGURE 2:**
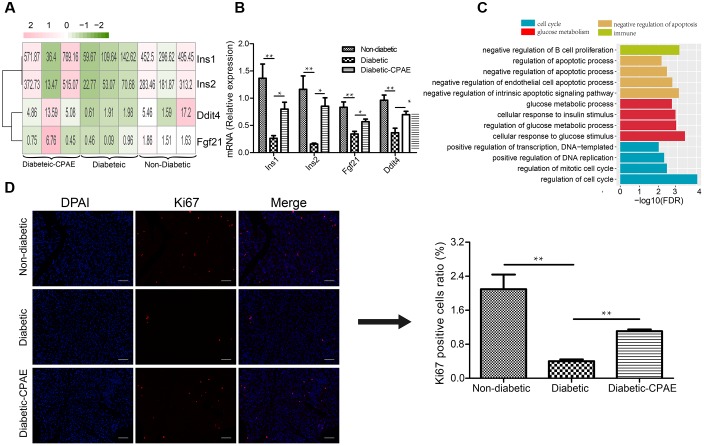
The expression levels and function enrichment of DEGs in pancreas tissue. **(A)** The expression levels of DEGs in pancreas tissues of three groups. Red: up-regulation; green: down-regulation, value: gene expression level (TPM). **(B)** Real-time qPCR analysis for gene expression (*n* = 6). **(C)** The gene ontology analysis of up-regulated DEGs in diabetic-CPAE-VS-diabetic group (*n* = 3). **(D)** Immunofluorescence analysis of Ki67 expression in pancreas tissues (magnification, 200×) and relative Ki67 positive cells in total pancreatic area (*n* = 3). The scale bar is 60 μm. ^∗^*p* < 0.05, ^∗∗^*p* < 0.001.

To investigate potential molecular mechanisms how CPAE affects diabetic islets, we performed transcriptome profiling analysis on the pancreas in three groups (non-diabetic, diabetic, diabetic-CAPE groups), respectively. Basic statistics for the sequenced samples were represented in **Supplementary Table S1**. Totally, 381 up-regulated and 343 down-regulated DEGs were found in the comparison of diabetic-VS- non-diabetic groups, while 153 up-regulated and 175 down-regulated DEGs were detected in the diabetic-CPAE-VS-diabetic group comparison (**Supplementary Figure S2** and **Supplementary Table S2**). Notably, expression levels of some crucial genes regulating functions and survival of pancreatic β-cell were dramatically changed with CPAE administration, such as *Ddit4*, *Fgf21* and insulin family members (**Figure 2A**). For example, the expression levels of *Ddit4*, *Fgf21* and *Ins1*, *Ins2* were markedly down-regulated in diabetic rats and up-regulated with CPAE administration (**Figure 2A**), and results of qRT-PCR experiments for these genes were consistent with the transcriptome profiling (**Figure 2B**). The expression levels of *Ins1* and *Ins2*, encoding preproinsulin, were highly correlated with the concentration of insulin *in vivo*, and *Ddit4* as a HIF-1-responsive gene may protect pancreatic β-cell from hypoxia and apoptosis, and *Fgf21* can lower blood glucose in STZ-induced insulin-deficient diabetic mice (Kim et al., 2015). These results suggested that CPAE could decrease damages in diabetic pancreas, eventually enhancing the synthesis of insulin and reducing excess blood glucose.

As previously concerned with the alteration of single important gene, functional enrichment analysis for DEGs could provide systematical clues to explore potential molecular mechanisms how CPAE exhibited protective effects on the diabetic pancreas. CPAE administration significantly up-regulated the expression of genes related to cell cycle processes, such as positive regulation of DNA replication, which showed the improvement of cell proliferation (**Figure 2C**). The results of immunofluorescence staining assay for pancreatic cells demonstrated the ratio of ki67 positive cells was dramatically reduced in diabetic group compared with other groups, whereas the ratio was increased in diabetic-CPAE group (**Figure 2D**), which indicated the CPAE could play positive effects on cell proliferation in diabetic pancreas. Meanwhile, pathway “the negative regulation of B cell proliferation” was up-regulated under CPAE treatment (**Figure 2C**), and levels of TNF-α and IL-6 were significantly decreased (**Figure 1E**), which implied a significant decrease of inflammation in the diabetic-CPAE group. In addition, terms related to glucose metabolism were up-regulated after CPAE administration, such as cellular response to glucose stimulus, cellular response to insulin stimulus (**Figure 2C**), which indicated an increase of glucose disposal in the pancreas. On the other hand, terms related to apoptosis were attenuated in the comparison of diabetic-CPAE-VS-diabetic group, such as up-regulated negative regulation of apoptotic process (**Figure 2C**). Furthermore, we found the CPAE decreased pathways including “Insulin resistance,” “Chemical carcinogenesis” and “Peroxisome” (**Supplementary Figure S3**).

### CPAE Alleviates Liver Injury by Improving Disordered Lipid Metabolism and Reducing Hepatic Steatosis, and Improves Diabetic Nephropathy

**FIGURE 3:**
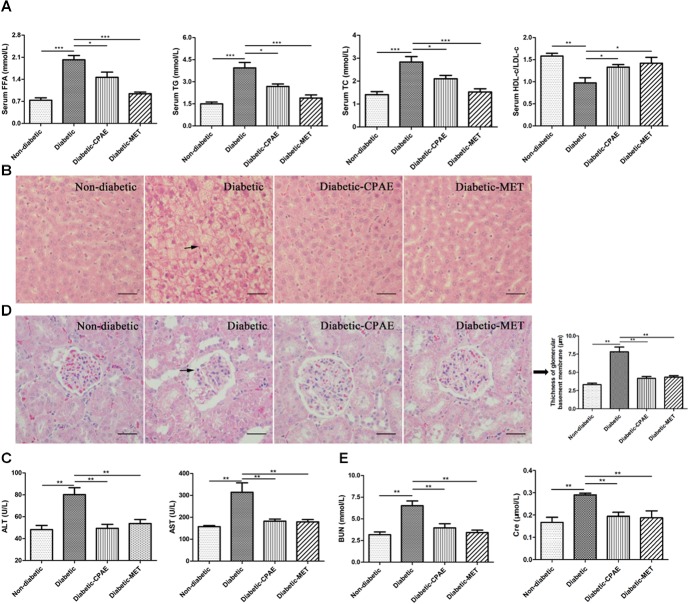
*Cyclocarya paliurus* leaves (CPAE) improves lipid metabolism disorders, reduces hepatic steatosis, attenuates liver injury and improves diabetic nephropathy. **(A)** Levels of FFA, TG, TC and HDL-c/LDL-c in the serum (*n* = 6). **(B)** Histopathological examination of liver tissues (magnification, 200×) (*n* = 3). The scale bar is 40 μm. **(C)** Levels of serum ALT and AST (*n* = 6). **(D)** Histopathological examination of kidney tissues (H&E staining) (magnification, 200×) and thickness of glomerular basement membrane (*n* = 3). The scale bar is 40 μm. **(E)** Levels of serum BUN and Cre (*n* = 6). ^∗^*p* < 0.05, ^∗∗^*p* < 0.001, ^∗∗∗^*p* < 0.0001.

The liver as a main metabolic organ plays important roles in diabetes, and diabetic nephropathy is recognized as a common complication of diabetes. Hence, we investigated the effects of CPAE on the liver and kidney in diabetic rats. Notably, the diabetic group exhibited serious dyslipidemia with significant increases of FFA, TG, TC and decrease of HDL-c/LDL-c in serum (**Figure 3A**), while the administration of CPAE or MET reversed the blood lipids close to normal levels (**Figure 3A**). Meanwhile, amounts of irregular vesicles of fat accumulation within hepatocytes (a typical pathological feature of hepatic steatosis) were observed in the diabetic group, while the CPAE or MET administration markedly alleviated fat accumulation (**Figure 3B**). Importantly, the levels of ALT and AST, as indicators of liver injury, were significantly down-regulated in the diabetic-CPAE or diabetic-MET groups and close to the normal level (**Figure 3C**), which indicated CPAE could help prevent liver injury of diabetic rats.

To further explore the influences of CPAE on the diabetic nephropathy, histological examination was performed for kidney sections. A thicker glomerular basement membrane was observed in diabetic rats compared with non-diabetic rats, while supplementation of CPAE or MET greatly decreased the thickness (**Figure 3D**). Meanwhile, we measured biochemical indicators of kidney function, and found that the levels of serum BUN and Cre were markedly elevated in diabetic rats (**Figure 3E**). Significantly, disordered elevations of BUN and Cre levels in diabetic rats were close to normal with CPAE or MET supplementation (**Figure 3E**). Combining results above, our data suggested that CPAE could prevent kidney from hyperglycemia, and ameliorate diabetic nephropathy.

### CPAE Protects Liver by Improving Impaired Lipid Metabolism and Reducing Oxidative Damage and Inflammation

**FIGURE 4:**
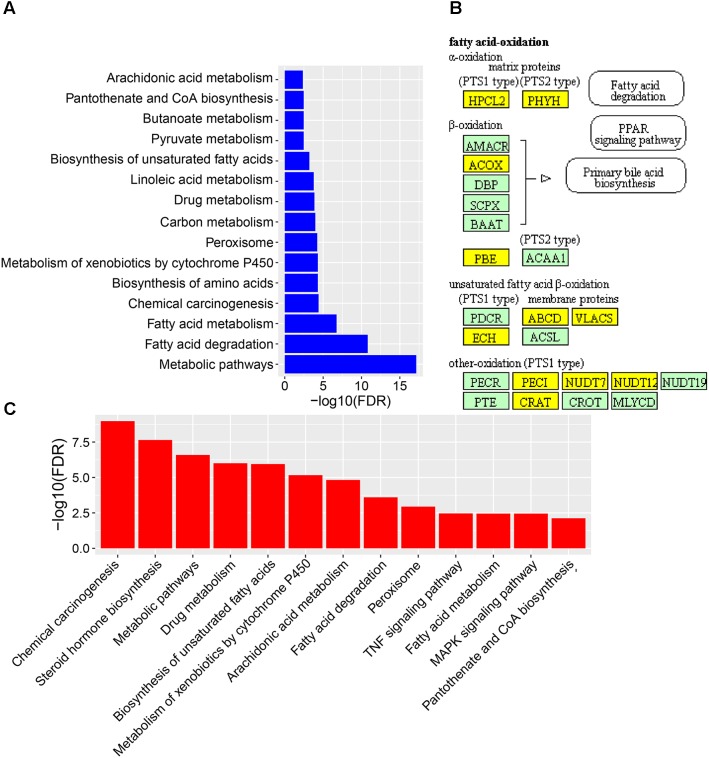
The expression levels and function enrichment of DEGs in liver tissue. **(A)** The functional enrichment of up-regulated DEGs in diabetic-VS-non-diabetic group (*n* = 3). **(B)** The fatty acids oxidation part in the peroxisome pathway enriched in up-regulated DEGs in diabetic-VS-non-diabetic group (*n* = 3). Yellow: up-regulated DEGs in diabetic-VS-non-diabetic; lightgreen: non-DEGs. **(C)** The functional enrichment of down-regulated DEGs in diabetic-CPAE–VS-diabetic group (*n* = 3).

To reveal the molecular mechanisms how CPAE affects the diabetic liver, we also performed transcriptome profiling analysis for livers from the three groups (**Supplementary Table S1**). We found 235 down-regulated and 677 up-regulated DEGs in the diabetic-VS-non-diabetic group comparison (**Supplementary Figure S2**). The up-regulated DEGs were mainly enriched in “Fatty acid degradation/metabolism,” “Metabolism of xenobiotics by cytochrome P450,” “Peroxisome and Chemical carcinogenesis pathway” (**Figure 4A**). The fatty acid degradation may be a homeostatic regulation of impaired glucose metabolism in rats, and a branch pathway related to fatty acids oxidation in the peroxisome was also significantly up-regulated (**Figure 4B** and **Supplementary Figure S4**). Besides, Metabolism of xenobiotics by cytochrome P450 as an important detoxification pathway in the liver was up-regulated to response to the damage of HFD&STZ on diabetic rats (**Figure 4A**), which may in turn caused oxidative stress (**Figure 1D**).

**FIGURE 5:**
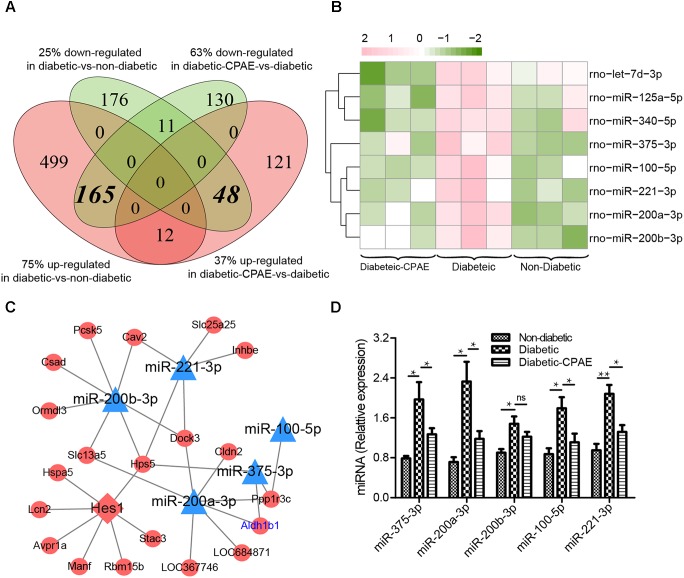
The expression levels and regulatory network of DEGs in liver tissues of three groups rat models. **(A)** The counts of DEGs in two comparisons for liver tissues (diabetic-VS-non-diabetic, diabetic-CPAE-VS-diabetic) (*n* = 3). **(B)** The expression levels of DEMs for both comparisons in liver tissues (*n* = 3). Red: up-regulation; green: down-regulation. **(C)** The miRNA-TF-Target regulatory network for liver tissues (*n* = 3). Triangle: miRNA; diamond: transcription factor; ellipse: gene; blue: up-regulation in diabetic-VS-non-diabetic and down-regulation in diabetic-CPAE-VS-diabetic; red: down-regulation in diabetic-VS-non-diabetic and up-regulation in diabetic-CPAE-VS-diabetic. **(D)** Real-time qPCR analysis for miRNA expression (*n* = 6). ^∗^*p* < 0.05, ^∗∗^*p* < 0.001.

CPAE administration significantly changed the expression levels of 487 genes compared with the diabetic group, including 181 up-regulated and 306 down-regulated DEGs (**Supplementary Figure S2**). Notably, most GO/KEGG terms enriched by DEGs in the comparison of diabetic-VS-non-diabetic groups, were also enriched by DEGs with the opposite expression tendency in the diabetic-CPAE-VS-diabetic group comparison (**Figures 4A,C**), such as “Fatty acid metabolism,” “Metabolism of xenobiotics by cytochrome P450,” “Peroxisome” and “Chemical carcinogenesis pathway.” These results implied the above biological processes may play vital roles for CPAE decreasing liver injuries induced by HFD&STZ. Meanwhile, the PPAR signaling pathway was up-regulated in the diabetic-CPAE group (**Supplementary Figure S5A**), which implied the CPAE may play the similar role as PPAR agonists (Goldstein et al., 2006). Furthermore, 165 DEGs were significantly up-regulated in the diabetic-VS-non-diabetic group comparison, which appeared the opposite trend in the diabetic-CPAE-VS-diabetic comparison; Simultaneously, 48 DEGs showed a contrary profiling, which were down-regulated in the diabetic-VS-non-diabetic comparison and up-regulated in the diabetic-CPAE-VS-diabetic groups (**Figure 5A**). In addition, crosstalk genes connecting multiple pathways in these DEGs may play important roles for maintaining liver function in CPAE group (**Supplementary Figure S5B**). For example, cytochrome P450 2e1 (*Cyp2e1*), the crosstalk gene for pathways “Chemical carcinogenesis” and “Metabolism of xenobiotics by cytochrome P450 pathways,” is an effective generator of reactive oxygen species (ROS) causing liver oxidative damage (Zhang et al., 2011), was up-regulated in the diabetic group but down-regulated in the diabetic-CPAE group.

Generally, miRNA and TF as important transcriptional regulators play vital roles in the pathogenesis of diabetes and diabetes-associated complications (Bierhaus et al., 2001; Baran-Gale et al., 2013). We detected 8 DEMs in the diabetic-CPAE-VS-diabetic comparison (**Figure 5B**), while 11 DEMs were found in the diabetic-VS-non-diabetic comparison. Most of the DEMs (5/8 in CPAE group) showed opposite expression tendency in the two comparisons (**Figure 5B**). To detailedly investigate potential regulatory mechanisms involved in the CPAE improving lipid metabolism, we constructed a miRNAs-TFs-genes network with the 5 DEMs (**Figure 5C**). MiR-200/375 combined with TF Hes1, as hubs (with much connections and degree) regulating 18 genes, played important roles in our network (**Figure 5C**). The previous study reported that miR-200 deficiency diminished hepatic steatosis and inflammation by reprogramming lipid and inflammation signaling pathways (Tran et al., 2017), and therapeutic inhibition of miR-375 can attenuate inflammatory response (Garikipati et al., 2017). Both miR-200 and miR-375 could targ etaldehyde dehydrogenase (*Aldh1b1*), which may in turn increase ROS and proinflammatory activities (Toye et al., 2007). Significantly, both transcriptome profiling and qRT-PCR experiments showed that the expression levels of miR-200 and miR-375 were down-regulated with CPAE administration (**Figures 5B–D**), which may result in the up-regulation of *Aldh1b1*, and thus increase ROS deactivation and decrease pro-inflammatory activities in diabetic-CPAE group. Meanwhile, CPAE administration also repressed the expression of miR-100 and miR-221, which were up-regulated in diabetic group (**Figure 5D**). Given above results, we referred that CPAE administration may reduce oxidative damage and inflammation in the diabetic liver through networks regulated by node miRNAs (miR-200/375/100/221, **Figure 5C**).

## Discussion

Pancreatic β-cell dysfunction and lipid metabolism disorders participated in the development and progression of diabetes. In this study, we have assessed the anti-diabetic effects of CPAE and explored the potential functional mechanisms. Our results revealed that CPAE exhibited anti-hyperglycemic and anti-hyperlipidemic effects on diabetic rats, which were consistent with previous studies (Wang et al., 2013; Xiao et al., 2017). Although MET was widely applied for diabetic therapy in clinic treatment, the side effects including lactic acidosis and hypoglycemia were reported by previous studies (Bodmer et al., 2008). Our results demonstrated that a low dose (200 mg/kg) of CPAE had analogous effects on hyperglycemia and some other indicators of diabetes compared to MET (**Figures 1–3**). Thus, CPAE may be a potential alternative hypoglycemic agent. Significantly, our data demonstrated CPAE could protect pancreas from diabetes caused damages through improving pancreatic β-cell function and survival, eventually enhancing the synthesis of insulin, thereby decreasing blood glucose. Meanwhile, CPAE administration could improve impaired lipid metabolism and reducing excessive oxidative damage in the liver.

Insulin is the only hypoglycemic hormone secreted by pancreatic β-cells. The dysfunction and excess loss β-cells will result in a decrease of insulin, which may lead to hyperglycemia and diabetes (Janikiewicz et al., 2015). Excessive oxidative stress could cause β-cell dysfunction (Lenzen, 2008), and long term HFD&STZ could induce serious oxidative stress (Torres et al., 1999; Thomas-Moya et al., 2008). Our biochemical results suggested that CPAE treatment significantly increased glutathione concentrations and SOD activities, and decreased levels of MDA (**Figure 1D**), which is consistent to the previous study (Wang et al., 2013). Furthermore, increased oxidative stress and simultaneous decline of antioxidant defense can lead to inflammation, damage of cellular organelle, and cell death (Newsholme et al., 2016). Our results demonstrated CPAE could markedly reduce serious inflammation and the damage of pancreas (**Figures 1C–E**). The increased insulin levels in diabetic-CPAE group may mainly attribute to two aspects: (1) elevated expression levels of *Ins1* and *Ins2*; (2) relatively increased β-cell mass. Our transcriptome and experiment data demonstrated that insulin genes were up-regulated with CPAE administration (**Figures 2A,B**). Meanwhile, CPAE administration up-regulated the expressions of *Ddit4* and *Fgf21* (**Figures 2A,B**), which were beneficial for pancreatic β-cell functions and survival (Wente et al., 2006). Furthermore, biological processes related to cell proliferation, such as DNA replication, were significantly up-regulated in diabetic-CPAE group (**Figure 2C**). Furthermore, the results of immunofluorence staining assay indicated the pancreas cells in diabetic-CPAE group presented higher activity for cell proliferation compared with the diabetic one (**Figure 2D**), which was consistent with transcriptome profiling analysis (**Figure 2C**). In addition, pathways “cellular response to glucose” and “insulin stimulus” were both enhanced in the pancreas with CPAE administration (**Figure 2C**). These results suggested that CPAE may increase the insulin levels through maintaining β-cell mass and up-regulating the expressions of insulin gene, which formed a positive feed loop and protected pancreatic β-cell, further in turn enhanced the functions and survival for pancreatic β-cell.

Long term HFD brings lipid metabolism disorders in the liver (Arcari et al., 2009), and STZ could further aggravate the process then cause serious injuries for the liver (Ogunyinka et al., 2017). Consistent with the previous study (Ma et al., 2015), our data indicated CPAE could ameliorate lipid metabolism disorders and dyslipidaemia (**Figures 3A,B**, **4B**). Transcriptome profiling indicated CPAE administration reduced oxidative stress and inflammation by repressing cytochrome P450 and enhancing fatty acid metabolism in diabetic rat liver (**Figure 4C** and **Supplementary Figure S5A**), which may contribute to the recovery and improvement of liver functions (Eid et al., 2009). Meanwhile, TF Hes1 was reported to control hepatic TG levels, here predicted regulating Hps5 (**Figure 5C**). Furthermore, CPAE significantly suppressed the expression level of miR-200 and miR-375 (**Figure 5B**), thereby enhanced the expressions of *Aldh1b1* and *Hps5*, which in turn protected the liver by reducing oxidative damage. The miR-200/375-*Aldh1b1/Hps5-Hes1* co-regulatory network may play positive roles for improving impaired lipid metabolism in diabetic liver. Combining these results above, we inferred that CPAE may reduce oxidative damage and ameliorate lipid metabolism disorders in diabetic rats, thereby restore normal functions of the liver for maintaining the balance of glucose and lipid metabolism.

In summary, our results showed that CPAE could ameliorate diabetes by protecting pancreatic β-cell from oxidative stress and inflammation, and maintaining the balance of glucose and lipid metabolism in the liver. Furthermore, transcriptome profiling and regulatory network analysis revealed potential mechanisms how CPAE affects diabetic rats, which will promote the studies of natural compounds for diabetes. Additionally, biochemical experiments combined with high-throughput technologies could provide compressive insights for exploring complex biological mechanisms underlying the efficacy of traditional Chinese medicine.

## Author Contributions

JL, ML, and QZ carried out the formal analysis and wrote the original draft. JL and MH carried out the experiment operation and sample preparation. YZ, XY, and A-YG supervised the project and acquired funding. QZ and YZ supervised the project and worked on the conceptualization, methodology, review, and editing.

## Conflict of Interest Statement

The authors declare that the research was conducted in the absence of any commercial or financial relationships that could be construed as a potential conflict of interest.

## Supplementary Material

FIGURE S1 **(A)** The level of FBG during all the experiment (*n* = 6). **(B)** The body weight dynamic change (*n* = 6). **(C)** The food intake during all the experiment (unit: g/day/rat) (*n* = 6). ^∗^Represents compared to non-diabetic group. #Represents compared to diabetic group. ^∗^*p* < 0.05, ^∗∗^*p* < 0.001, ^∗∗∗^*p* < 0.0001. ^#^*p* < 0.05, ^##^*p* < 0.001. ns represented no significance.

FIGURE S2 Counts of DEG and DEMs in different comparisons for liver and pancreas (*n* = 3).

FIGURE S3 The gene ontology analysis of down-regulated DEGs in CPAE administration (*n* = 3).

FIGURE S4 The enriched peroxisome pathway for up-regulated DEGs in diabetic-VS-non-diabetic (*n* = 3). Yellow: up-regulated DEGs in diabetic-VS-non-diabetic; lightgreen: non-DEGs.

FIGURE S5 **(A)** The enriched pathways for up-regulated DEGs in diabetic-CPAE-VS-diabetic. **(B)** The KEGG crosstalk network of DEGs down-regulated in diabetic and up-regulated in diabetic-CPAE. Small node: DEGs; large node: KEGG PATHWAY.

TABLE S1 Samples sequenced on small RNA-seq and RNA-seq platform.

TABLE S2 Differentially expressed genes (DEGs) and miRNAs (DEMs) in pancreas and liver (*n* = 3).
