# Supplementary figures and images for: Investigating the Molecular Mechanism of Aqueous Extract of Cyclocarya paliurus on Ameliorating Diabetes by Transcriptome Profiling
Source: Front Pharmacol. 2018 Aug 9;9:912. doi: 10.3389/fphar.2018.00912 (PMC6095059; doi:10.3389/fphar.2018.00912)

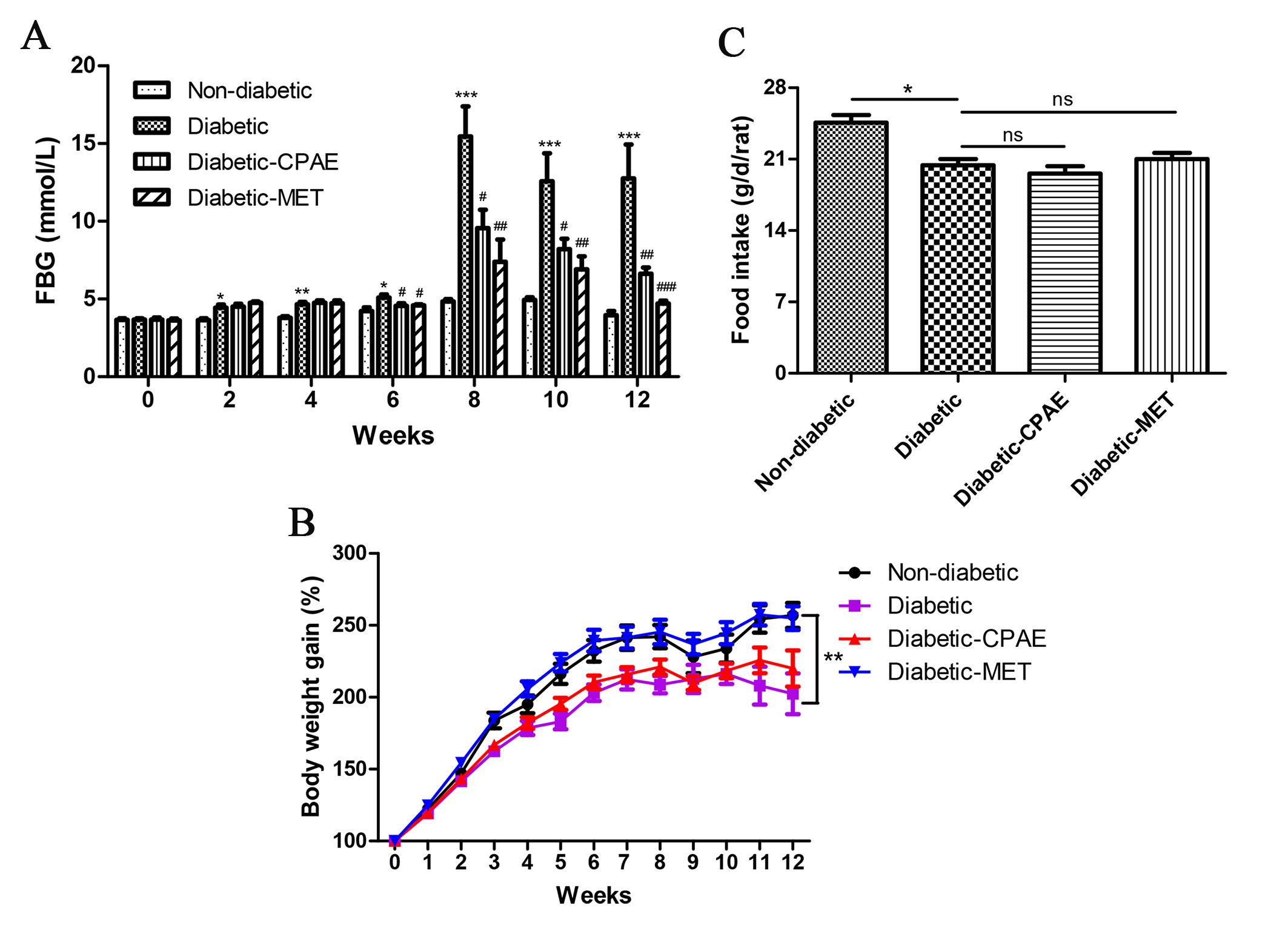

Supplement: FIGURE S1 — (A) The level of FBG during all the experiment (n = 6). (B) The body weight dynamic change (n = 6). (C) The food intake during all the experiment (unit: g/day/rat) (n = 6). ∗Represents compared to non-diabetic group. #Represents compared to diabetic group. ∗p < 0.05, ∗∗p < 0.001, ∗∗∗p < 0.0001. #p < 0.05, ##p < 0.001. ns represented no significance. [file Image_1.TIF]

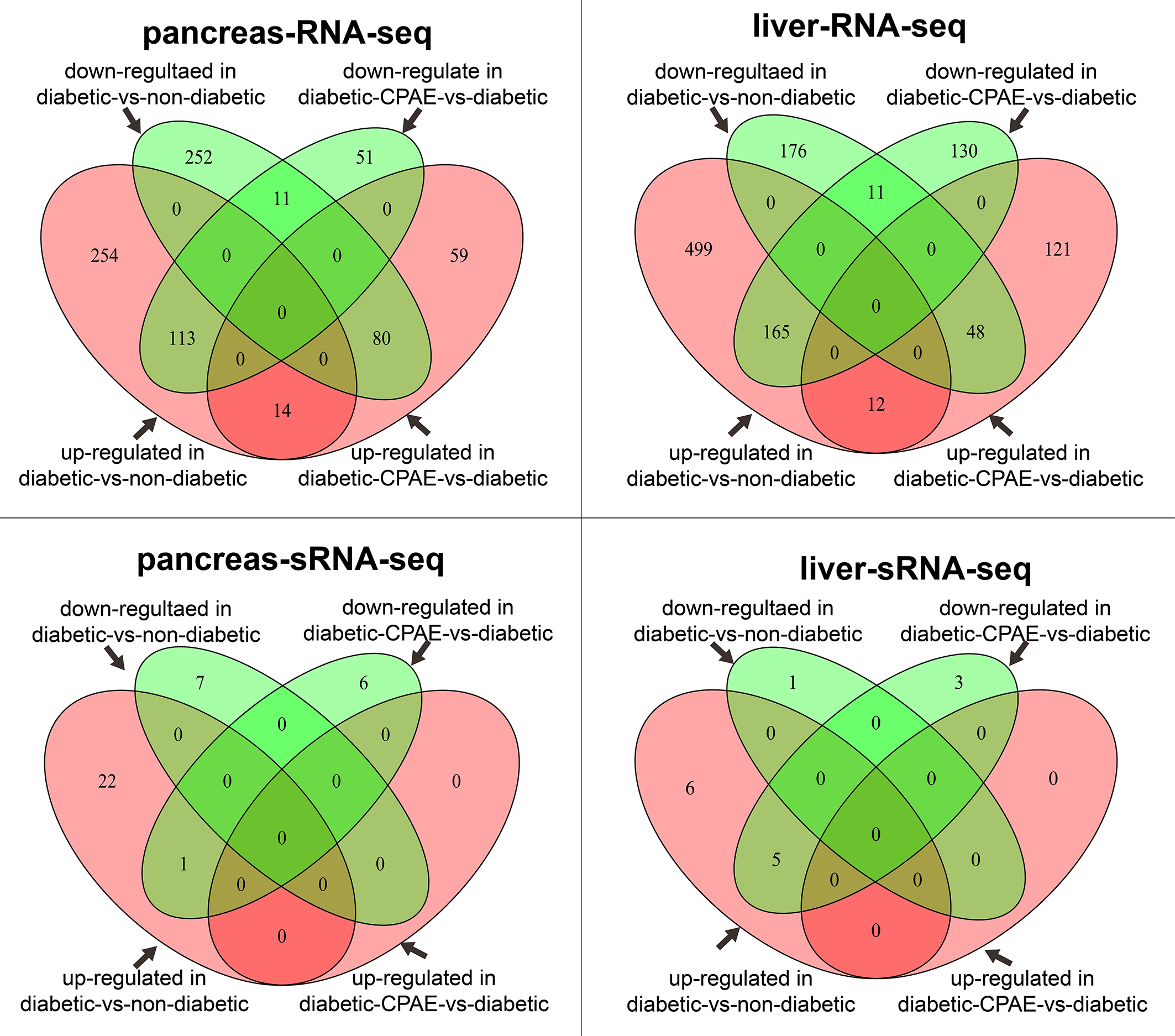

Supplement: FIGURE S2 — Counts of DEG and DEMs in different comparisons for liver and pancreas (n = 3). [file Image_2.TIF]

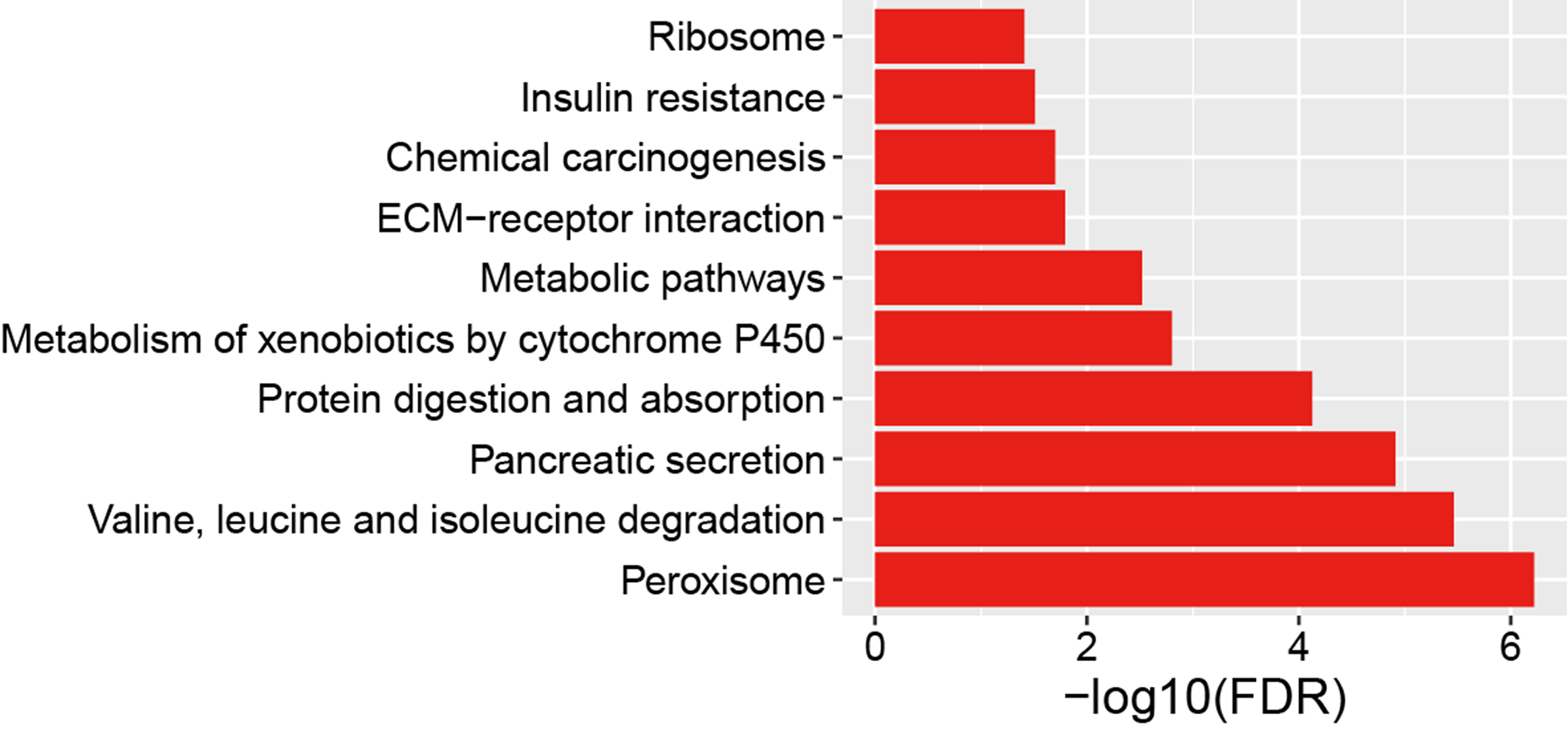

Supplement: FIGURE S3 — The gene ontology analysis of down-regulated DEGs in CPAE administration (n = 3). [file Image_3.TIF]

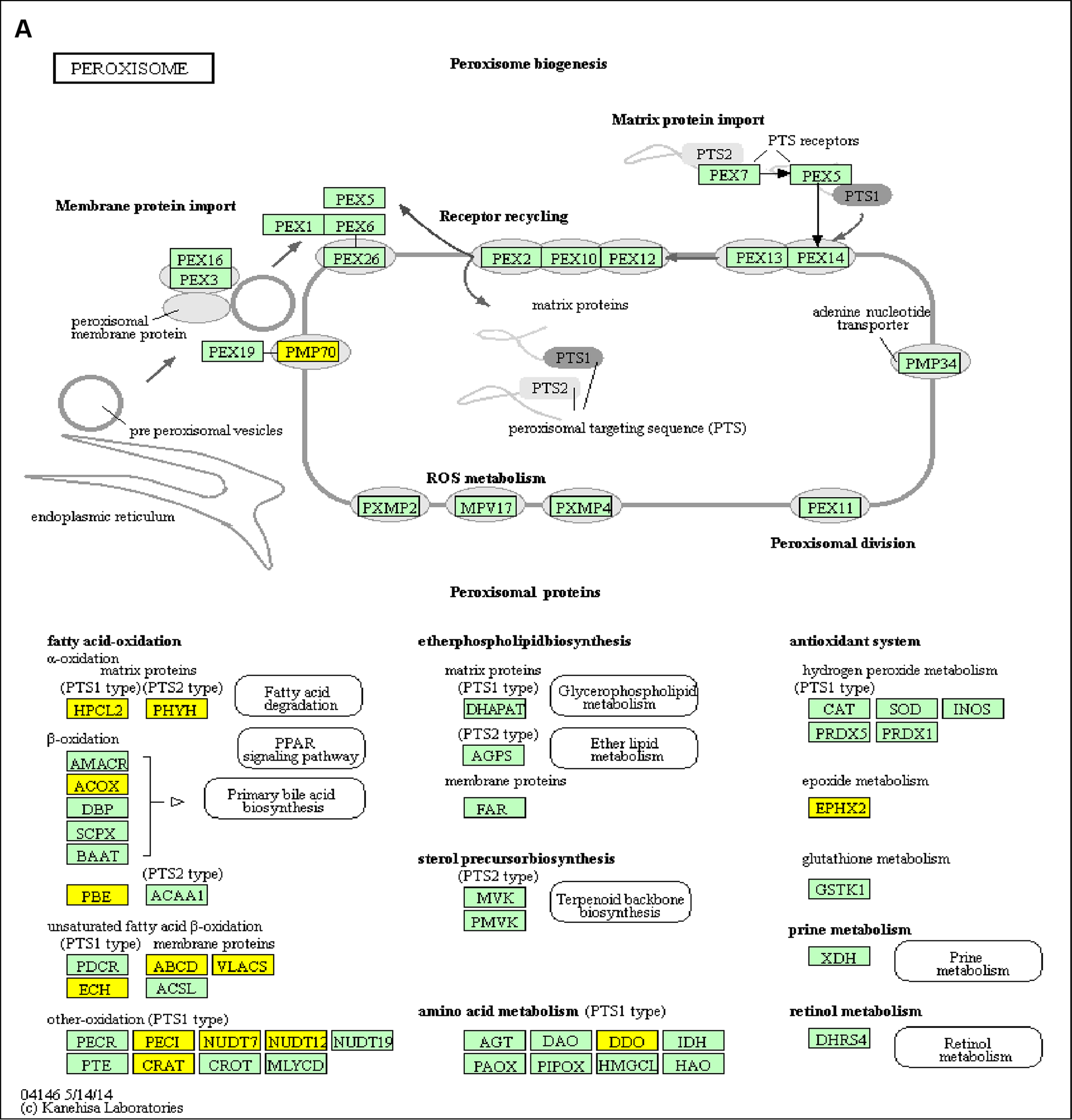

Supplement: FIGURE S4 — The enriched peroxisome pathway for up-regulated DEGs in diabetic-VS-non-diabetic (n = 3). Yellow: up-regulated DEGs in diabetic-VS-non-diabetic; lightgreen: non-DEGs. [file Image_4.TIF]

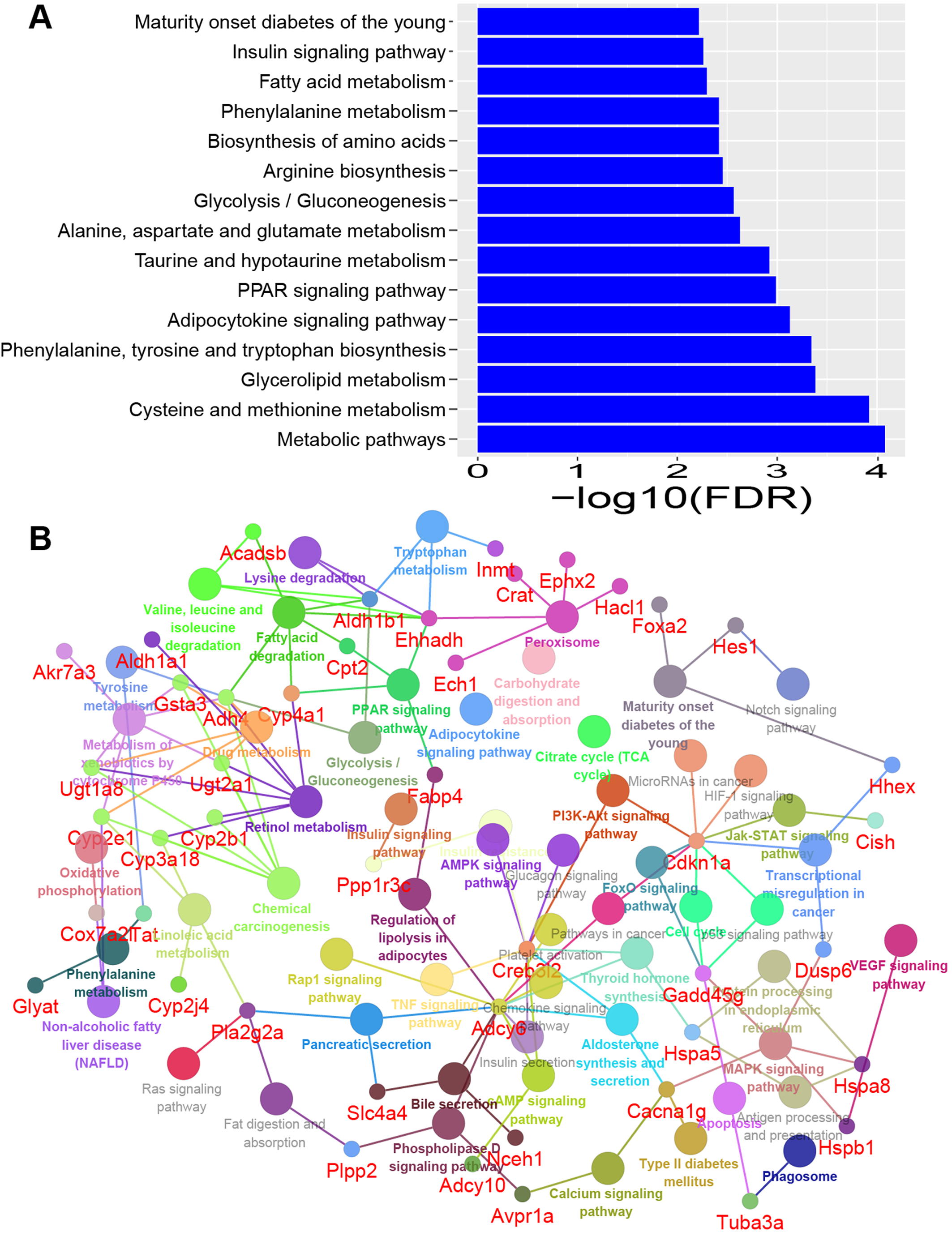

Supplement: FIGURE S5 — (A) The enriched pathways for up-regulated DEGs in diabetic-CPAE-VS-diabetic. (B) The KEGG crosstalk network of DEGs down-regulated in diabetic and up-regulated in diabetic-CPAE. Small node: DEGs; large node: KEGG PATHWAY. [file Image_5.TIF]
