# Supplementary material for: Investigating the Molecular Mechanism of Aqueous Extract of Cyclocarya paliurus on Ameliorating Diabetes by Transcriptome Profiling
Source: Front Pharmacol. 2018 Aug 9;9:912. doi: 10.3389/fphar.2018.00912 (PMC6095059; doi:10.3389/fphar.2018.00912)
Supplement: TABLE S1 — Samples sequenced on small RNA-seq and RNA-seq platform. [file Table_1.DOCX]

**Table 1** Samples sequenced on small RNA-seq and RNA-seq platform.

| **Issue** | **Platform** | **Non-diabetic** | | | **Diabetic** | | | **Diabetic-CPAE** | | |
| --- | --- | --- | --- | --- | --- | --- | --- | --- | --- | --- |
| **Liver** | mRNA | L-A3A | L-A4A | L-A7A | L-B3A | L-B4A | L-B7A | L-C1A | L-C2A | L-C3A |
|  | sRNA | L-A3A | L-A4A | L-A7A | L-B3A | L-B4A | L-B7A | L-C1A | L-C2A | L-C3A |
| **Pancreas** | mRNA | A1-NA | A2-LA | A6-NA | P-B3B | P-B4B | P-B7B | P-C1B | P-C2B | P-C3B |
|  | sRNA | A1-NA | A2-LA | A6-NA | P-B3B | P-B4B | P-B7B | P-C1B | P-C2B | P-C3B |
